# Multiple time points of transcriptome analysis revealed altered genes involved in maintaining hibernation in the hypothalamus of *Tamias sibiricus*

**DOI:** 10.3389/fnins.2024.1501223

**Published:** 2024-12-23

**Authors:** Tian Zhang, Chao Yang, Yaxiu Guo, Zihan Xu, Minbo Zhao, Feng Wu, Hongyu Zhang, Hailong Wang, Xiukun Sui, Siyu Jiang, Rongqiao He, Zhongquan Dai, Ying Liu, Yinghui Li

**Affiliations:** ^1^School of Life Sciences, Beijing University of Chinese Medicine, Beijing, China; ^2^National Key Laboratory of Space Medicine, China Astronaut Research and Training Center, Beijing, China; ^3^School of Life Science and Technology, Harbin Institute of Technology, Harbin, China; ^4^Department of Pathology and Forensics, Dalian Medical University, Beijing, China; ^5^State Key Laboratory of Brain and Cognitive Science, Institute of Biophysics, Chinese Academy of Sciences, Beijing, China

**Keywords:** hibernation bouts, sodium ion channel, potassium ion channel, glutamate receptor, torpor

## Abstract

Hibernation, an adaptive mechanism to extreme environmental conditions, is prevalent among mammals. Its main characteristics include reduced body temperature and metabolic rate. However, the mechanisms by which hibernating animals re-enter deep sleep during the euthermic phase to sustain hibernation remain poorly understood. We selected the *Tamias sibiricus* as a model organism and conducted transcriptomic sequencing of its hypothalamus at multiple time points throughout hibernation. Through the strategies of gene set filtering and intersection analysis, we effectively filtered out redundant data, identifying a subset of genes whose expression was downregulated during the euthermic phase potentially inducing re-enter deep sleep, thereby maintaining the periodic cycles of torpor and arousal. These cycles are crucial for sustaining the overall hibernation process. Notably, genes associated with sodium and potassium ion channels were significantly enriched. Specifically, potassium ion-related genes such as Kcnc1, Kcna2, Kcng4, and Kcna6, along with sodium ion-related genes such as Scn1a and Hcn2, were markedly downregulated. qRT-PCR validation of four of these genes (Kcnc1, Kcna6, Scn1a, and Hcn2) confirmed significant downregulation during the euthermic phase compared to the deep sleep phase, further supporting our transcriptomic findings. This study provides novel insights into the hypothalamic transcriptome dynamics at various hibernation stages. Although the functional roles of these genes require further investigation, our findings lay the groundwork for future studies to elucidate the molecular mechanisms underlying hibernation.

## 1 Introduction

Hibernation is a widespread adaptive mechanism among mammals, enabling them to survive extreme environmental conditions by significantly decreasing their body temperature and metabolic rate. For example, small hibernating mammals can reduce their body temperature to 4–10°C and lower their metabolic rates to 1–2% of normal levels (Ruf and Geiser, 2015; Heldmaier et al., 2004; Jastroch et al., 2016). Periodic cycles of torpor and arousal during hibernation help mitigate potential damage from prolonged inactivity (Rice et al., 2020; Cerri et al., 2016). Remarkably, hibernating animals exhibit reduced bone loss, muscle atrophy, and enhanced resistance to radiation damage. These adaptations have significant implications for human health, particularly in long-term space exploration, where inducing hibernation-like states could reduce energy consumption and mitigate the adverse effects of weightlessness on skeletal and muscular systems (Choukér et al., 2021; Choukèr et al., 2019). Understanding these natural adaptations provides a foundation for exploring synthetic hibernation in non-hibernating species.

Recent advancements have made it feasible to induce synthetic torpor, a hibernation-like state, in non-hibernating species through specific methods (Shi et al., 2021; Cerri et al., 2013; Eisner et al., 2017; Soto et al., 2018; Zhang et al., 2023). However, most studies in this area have been conducted using fasting-induced torpor models in mice and rats. To bridge this gap, understanding the mechanisms underlying natural hibernation in mammals could provide valuable insights for developing synthetic torpor in humans. Currently, the main animal models used in hibernation research include Arctic ground squirrels, thirteen-lined ground squirrels, European hamsters, and Siberian hamsters. These animals exhibit regional distribution patterns which can pose challenges for researchers. For instance, the Arctic ground squirrel (*Urocitellus parryii*) is primarily found in parts of the United States and Canada, while thirteen-lined ground squirrels (*Ictidomys tridecemlineatus*) inhabit a broad range of North America, including northern territories with extended winters (Karels and Boonstra, 2000; Chmura et al., 2023; Armstrong, 1971; Merriman et al., 2012; Vaughan et al., 2006). European hamsters (*Cricetus cricetus*) are mainly distributed in central Russia, Crimea, the Ural region, and northern Kazakhstan (Feoktistova et al., 2017). Accessing these animals can be difficult for researchers outside these regions.

In contrast, the Siberian chipmunk (*Tamias sibiricus*) has a wider distribution, spanning Japan, Russia, Kazakhstan, North Korea, South Korea, Mongolia, and China. This species has been studied for its seed storage behavior, parvovirus transmission, and specific hibernation patterns (Li et al., 2022; Zhang et al., 2016; Kim et al., 2022; Zhou et al., 2022; Kondo et al., 2006; Tsukamoto et al., 2019). The widespread availability of *T. sibiricus* makes it a practical and valuable model for hibernation research. *T. sibiricus* exhibits a food-storing strategy, accumulating food supplies in various locations to ensure adequate nutrition during the winter months. In the wild, *T. sibiricus* typically hibernates in burrows or tree cavities that provide stable low temperatures and darkness, essential for maintaining their hibernation state (Marmet et al., 2009; Horii et al., 2018). Considering these natural conditions, we established a controlled laboratory environment to mimic the hibernation conditions of *T. sibiricus* (details provided in Methods). This approach aims to reflect the natural hibernation conditions and assess whether these laboratory-induced conditions can accurately represent the characteristics observed in the wild.

A hibernation bout refers to the periodic cycles of torpor and arousal during hibernation. Recent research has primarily focused on the mechanisms that induce mammals to enter hibernation (Cerri et al., 2013; Yang et al., 2023; Tupone et al., 2013). These studies have revealed various physiological and molecular triggers that initiate the hibernation state. However, there is relatively little research on the mechanisms that prompt hibernating animals to spontaneously re-enter deep sleep during the euthermic phase to sustain hibernation, under stable environmental conditions, rather than exiting hibernation entirely and returning to a normal state. Understanding these mechanisms is crucial for a comprehensive understanding of hibernation and could provide insights into maintaining synthetic torpor in non-hibernating species.

The hypothalamus is an important center for regulating the balance of energy metabolism, eating behavior, and sleep behavior in mammals (Chen et al., 2018; Harrold et al., 1998; Bao et al., 2019). Recent studies have highlighted its crucial role in regulating hibernation. During hibernation, the hypothalamus undergoes seasonal changes in endogenous neuropeptides (Mousavi et al., 2023). Research on thirteen-lined ground squirrels has shown that specific extracellular matrix (ECM) structures called perineuronal nets (PNNs) in the hypothalamus undergo changes during hibernation, interbout arousals (IBA), and non-hibernating periods. Three regions in the hypothalamus with PNNs, including the ventrolateral hypothalamic area, paraventricular nucleus (PVN), and anterior hypothalamic area, have been identified as crucial for limiting plasticity and maintaining basic brain connectivity during hibernation (Marchand and Schwartz, 2020). Different ɑ, *β*, and *γ* subunits operate as major elements either at the onset of torpor or during the induction of the arousal state in the Syrian golden hamster (*Mesocricetus auratus*) (Alò et al., 2010). Examination of the Phodopus genome in combination with transcriptome sequencing of the hamster diencephalon under winter and summer conditions further supports the crucial role of the hypothalamus in hibernation (Bao et al., 2019). In conclusion, the hypothalamus plays a crucial role in the hibernation process of hibernating animals. Therefore, we hypothesize that during mammalian hibernation, there are regulatory mechanisms within the hypothalamus that enable mammals to re-enter deep sleep during the euthermic phase to sustain hibernation.

In this study, we utilized the *T. sibiricus* as a model organism and adopted five groups of time points: the pre-hibernation active phase (ACT), mid-hibernation deep sleep phase (M-DS), mid-hibernation euthermia phase (M-EUT), end-of-hibernation deep sleep phase (E-DS), and end-of-hibernation euthermia phase (E-EUT). We conducted transcriptomic sequencing analysis of the hypothalamus of *T. sibiricus* at each time point. During the mid-hibernation arousal period, it was discovered that the expression levels of hypothalamic ion channel-related genes in *T. sibiricus* were notably lower than those during the mid-hibernation deep sleep phase, suggesting that these reduced levels may be crucial for the ability to re-enter hibernation cycles.

## 2 Materials and methods

### 2.1 Animal experiment

Adult male *T. sibiricus* (18 months old; weight 90–120 g) were housed under controlled experimental conditions in the Animal Laboratory of the China Astronaut Research and Training Center. The initial environment was maintained at 22°C with 40% humidity and a 12 h:12 h light: dark photoperiod. The animals were provided with a diet of mice maintenance food and sunflower seeds, along with water available *ad libitum*. After a two-month fattening period, the *T. sibiricus* were individually housed in cages and transitioned to a 24-h dark environment for the hibernation experiment. The experimental room temperature was regulated at 10 ± 2°C with relative humidity maintained at 40 ± 10%. For this study, a total of 15 samples were collected, with three biological replicates in each of the five groups: ACT, M-DS, M-EUT, E-DS, and E-EUT.

**Figure 1:**
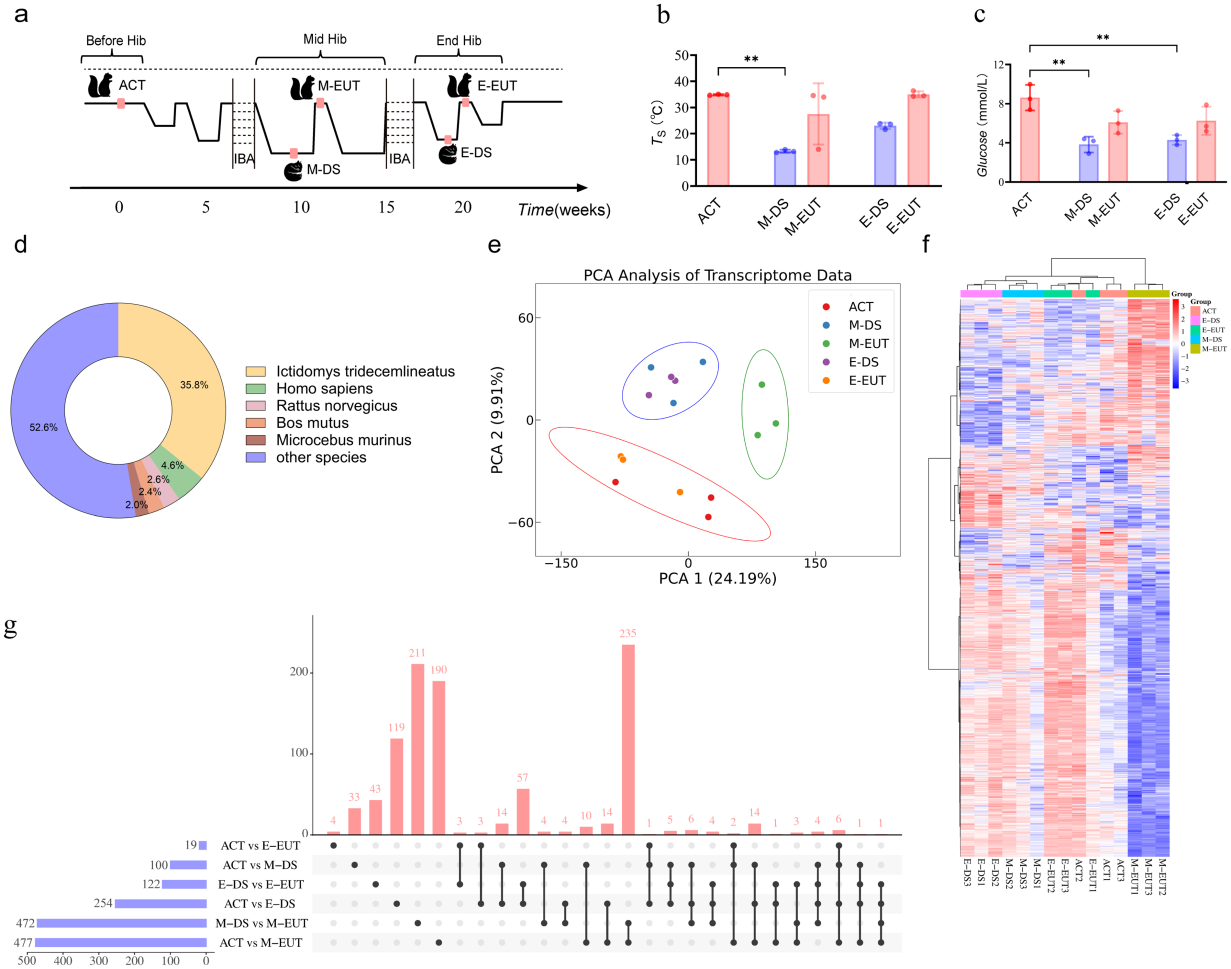
Sampling process and transcriptomics analysis of hibernation in *T. sibiricus*. **(A)** Schematic representation of the sampling process for *T. sibiricus*. Before Hib, Mid Hib, and End Hib represent the pre-hibernation, mid-hibernation, and end-of-hibernation phases, respectively. The IBA represents the periodic arousal processes occurring throughout the hibernation cycle, of which several cycles are omitted in the schematic for simplicity. **(B)** Comparison of body temperature across different hibernation states (ACT, M-DS, M-EUT, E-DS, E-EUT) (*n* = 3 for each group). The red bars represent the arousal states (ACT, M-EUT, E-EUT), while the blue bars represent the deep sleep states (M-DS, E-DS). Each bar shows the mean T _S_ with error bars indicating the SD. Significant differences were observed between ACT and M-DS (**p < 0.01). **(C)** Comparison of blood glucose levels (Glucose, mmol/L) across different hibernation states (ACT, M-DS, M-EUT, E-DS, E-EUT) (*n* = 3 for each group). The red bars represent the arousal states (ACT, M-EUT, E-EUT), while the blue bars represent the deep sleep states (M-DS, E-DS). Each bar shows the mean glucose level with error bars indicating the SD. Significant differences were observed between ACT and M-DS, and ACT and E-DS (**p < 0.01). **(D)** Distribution of transcript sequence similarity based on the Nr database. **(E)** PCA score plots from the hypothalamus after preprocessing and normalization by EigenMS. For the hypothalamus, 15 animals were analyzed from ACT, M-DS, E-EUT, E-DS, and E-EUT (*n* = 3) (circle represents the 95% confidence interval). **(F)** bidirectional complete linkage clustering heatmap based on RNA-Seq analysis of the hypothalamus of *T. sibiricus*. Rows indicate individual transcripts significantly upregulated (red) or downregulated (blue) at the group level (FDR < 0.05). Columns represent individual *T. sibiricus* (biological replicates; *n* = 3). **(G)** The upset plot shows the distribution and intersections of differentially expressed genes (DEGs) across six groups (ACT vs. M-DS, ACT vs. M-EUT, M-DS vs. M-EUT, ACT vs. E-DS, ACT vs. E-EUT, E-DS vs. E-EUT). The horizontal bars on the left represent the total number of DEGs identified in each individual group comparison, with blue bars indicating the specific DEG counts. The vertical bars above represent the number of shared DEGs across different combinations of groups, as indicated by the connected black dots below each bar. The red vertical bars highlight the number of intersecting DEGs, indicating the degree of overlap among the groups. **(H)** Top 20 KEGG pathways enriched for downregulated differentially expressed genes (DEGs) between the ACT and M-EUT groups. Pathways are ranked by-log10 (adjusted *p*-value) significance. The x-axis represents the enrichment score, calculated as-log10 (p-value), and the y-axis lists the pathway names. The color gradient, transitioning from blue to red, indicates increasing significance, with red representing higher-log10 (adjusted p-value). The size of the circles represents the number of genes enriched in each pathway.

To confirm the hibernation state, we monitored surface body temperature using a thermal imaging camera (Fortic 800, China). Due to the continuous dark environment, the circadian rhythms of *T. sibiricus* during hibernation followed an endogenous cycle, independent of a traditional 24-h sleep–wake cycle. The torpor-arousal cycles naturally varied, with each cycle typically lasting 3–14 days (Mohr et al., 2020). A sustained low body temperature over several hours and the absence of movement were used as indicators of hibernation onset (Staples, 2014). The entire hibernation process starts in mid-November and lasts until the end of March the following year, spanning a total of 20 weeks. We collected the first sampling point (ACT) before *T. sibiricus* entered hibernation in November. Two mid-hibernation sampling points (M-DS and M-EUT) were collected in January of the following year, around the 10th week. The final two end-of-hibernation sampling points (E-DS and E-EUT) were collected at the end of March, during the *T. sibiricus’* last re-entry into torpor. The criteria for determining whether *T. sibiricus* was in an arousal state or torpor state were as follows: regardless of whether it was mid-or end-hibernation, *T. sibiricus* remaining in a torpor state for more than 36 h was considered to be in a deep sleep phase, specifically the mid-hibernation deep sleep phase (M-DS) or end-of-hibernation deep sleep phase (E-DS). In contrast, *T. sibiricus* staying in an arousal state for more than 8 h was identified as being in the mid-hibernation euthermia phase (M-EUT) or end-of-hibernation euthermia phase (E-EUT). For consistency, we defined the onset of torpor and arousal as time zero (0 h) and sampled hypothalamic tissues 1–2 h after each animal maintained a torpor state for over 36 h or an arousal state for over 8 h. This timing allowed us to accurately capture gene expression changes associated with the specific physiological states of torpor and arousal (Figures 1A,B). Hypothalamic tissues were then collected at these points to ensure consistency for subsequent RNA extraction and analysis. These time points were chosen to capture the dynamic changes in gene expression associated with different stages of the hibernation cycle. Body weight was measured at each sampling point (ACT, M-DS, M-EUT, E-DS, E-EUT). Blood glucose levels were determined using Roche blood glucose test strips.

All animal research was conducted in accordance with the ARRIVE guidelines and approved by the Institutional Animal Care and Use Committee of the China Astronaut Research and Training Center. The study complied with local legislation and institutional requirements.

### 2.2 RNA extraction and transcriptome sequencing

Total RNA was isolated by TRIzol reagent (Invitrogen, United States) according to the manufacturer’s instructions. To ensure stringent quality control, the integrity of the RNA was meticulously assessed using an Agilent 2,100 Bioanalyzer. The synthesis of the first strand of cDNA was accomplished in the M-MuLV reverse transcriptase reaction system, employing fragmented messenger RNA as a template and random oligonucleotides as primers. This was followed by the degradation of the RNA with RNase H and the subsequent synthesis of the second strand of cDNA using DNA Polymerase I, with deoxyribonucleotide triphosphates (dNTPs) serving as the raw material.

The resultant double-stranded cDNA was purified and subjected to end repair, the addition of an A-tail, and the ligation of sequencing adapters. After these modifications, the cDNA fragments were size-selected to target approximately 250-300 bp using AMPure XP beads. The selected cDNA fragments were then amplified via PCR to produce the final library, which was further purified using AMPure XP beads.

Once the library passed quality control measures, it was pooled with other libraries based on their effective concentration and the data output requirements for downstream analysis. The pooled libraries were sequenced using the Illumina NovaSeq 6,000 platform. The raw image data generated by the high-throughput sequencer were processed into sequence reads by CASAVA software, which performs base calling. Subsequently, the original data underwent filtering, error rate verification, and GC content distribution analysis to obtain high-quality reads for subsequent analytical procedures.

### 2.3 Functional annotation

In the present investigation, a reference genome-independent analytical approach was employed. Post-acquisition of high-quality sequence reads Trinity version was utilized to assemble these reads *de novo* (Grabherr et al., 2011). Subsequently, Corset, as described by Nadia M. Davidson and Alicia Oshlack in 2014, was applied to create clusters based on shared reads among transcripts, integrating information on transcript expression levels across various samples with the Hierarchical Clustering algorithm (H-Cluster), thereby segregating differentially expressed transcripts into distinct clusters (Davidson and Oshlack, 2014). Each cluster was then designated as a “gene.” Functional annotation of the genes was conducted to acquire comprehensive data from databases including Nr, Nt, Pfam, KOG/COG, Swiss-Prot, KEGG, and GO.

### 2.4 Venn, PCA, and heatmaps analysis

Linear regression analysis was performed using the stats.linregress package from SciPy v1.14.1 (Virtanen et al., 2020). The matplotlib_venn package was used to generate the Venn diagrams (Tretyakov, 2012). Principal component analysis (PCA) was conducted with the sklearn.decomposition.PCA package and cluster analysis were carried out using the sklearn.cluster.KMeans package from scikit-learn library (Fabian Pedregosa, 2011). Data standardization was achieved with the sklearn.preprocessing.StandardScaler package (Fabian Pedregosa, 2011).Visualization of linear regression, PCA, and scatterplots in heatmaps was facilitated by the seaborn.scatterplot package from the seaborn library (Waskom, 2021). Heatmap clustering was performed using a bidirectional complete linkage clustering method, implemented with the scipy.cluster.hierarchy.linkage function from the SciPy library (Virtanen et al., 2020). All the above analyses were carried out using Python 3.0.

### 2.5 GO and KEGG analysis

Differential expression analysis was performed using the DESeq2 statistical model, which directly takes raw read counts as input (Love et al., 2014). This approach ensures accurate normalization and identification of differentially expressed genes (DEGs). Genes with adjusted *p*-value <0.05 and a threshold fold change were considered significantly differentially expressed. To elucidate the functional significance of these DEGs, Gene Ontology (GO) enrichment analysis was conducted using the GOseq tool, which adjusts for gene length bias. Additionally, KEGG pathway enrichment analysis was performed using KOBAS software (Kanehisa et al., 2008; Young et al., 2010).

### 2.6 Genome alignment method

To determine the origin of the sequencing fragments, which are randomly fragmented mRNA, it is essential to map the cleaned reads to a reference genome. This process allows for the identification of the specific genes from which the reads are transcribed. In our study, we used the HISAT2 alignment tool for this purpose (Kim et al., 2015). HISAT2, developed by a lab at Johns Hopkins University, employs both global and local search strategies for RNA-seq read alignment. It utilizes a large FM-index that spans the entire genome, enabling rapid and accurate mapping of RNA-seq reads to the reference genome. The software is optimized for spliced read alignment, making it particularly suitable for processing transcriptomic data where reads span across exon-exon junctions. The resulting mapped reads were subsequently analyzed to associate the fragments with their respective genes, allowing for the identification of transcriptional events and alternative splicing patterns. This approach ensured high alignment accuracy and coverage for the RNA-seq data analysis in our study.

### 2.7 Total RNA extraction and cDNA synthesis and quantitative qRT-PCR analysis

Tissue samples were lysed in TRIzol (Invitrogen, United States). Quality analysis of these RNA samples was performed on a Nanodrop 2000. RNA samples extracted from the time-course samples were used to prepare 1 μg cDNA libraries using a High-Capacity cDNA Reverse Transcription Kit (TAKARA, China), following the manufacturer’s instructions. Gene expressions were quantified with a validated SYBR Green Master Mix (TAKARA, China) on a quantitative real-time PCR machine and Ct values were calculated using Quant studio real-time PCR. Relative gene expressions were computed using the 2^-ΔΔCt method. All the primers used in qRT-PCR are listed in Supplementary Table S1.

### 2.8 Statistical analysis

Statistical analyses were conducted using GraphPad Prism 9.5.1. An ordinary one-way ANOVA with multiple comparisons was applied to assess differences in blood glucose levels, body temperature, and PCR validation results. Significance levels are indicated as follows: *p < 0.05, **p < 0.01, and ****p < 0.0001, with specific *p*-values noted in the figure legends as appropriate.

### 2.9 Data availability

We acknowledge that the transcriptomic datasets referenced in the manuscript are available in the NCBI Sequence Read Archive (SRA) repository, BioProject PRJNA1101579, as stated. However, the genomic data referenced in this study is still under review and therefore not yet publicly available. The project ID for the genomic data is PRJNA1192718, and it will be accessible through the NCBI repository once the review process is complete.

## 3 Result

### 3.1 Initial data collection and transcriptome preprocessing

To comprehensively observe the differences in gene expression in the hypothalamus throughout the hibernation bouts of *T. sibiricus* under different torpor and arousal states, we selected a total of 15 samples, with three biological replicates (*n* = 3) for each of the five distinct and representative time points across the entire hibernation period: ACT, M-DS, M-EUT, E-DS, and E-EUT states (Figure 1A). Unlike previous studies, which often focus on only three time points—before, during, or after hibernation—we expanded the sampling to include both mid-and late-hibernation stages, capturing both torpor and aroused states at each stage. This comprehensive approach enables a more in-depth analysis of the molecular dynamics of hibernation, providing a richer dataset for downstream analyses and data mining. We conducted a differential analysis of body temperature and blood glucose levels across the five hibernation states (ACT, M-EUT, E-EUT, M-DS, and E-DS). Statistically significant differences in body temperature were observed, with M-DS exhibiting significantly lower body temperature compared to ACT (*p* < 0.01; Figure 1B). For blood glucose levels, significant differences were found, with both M-DS and E-DS showing significantly lower glucose levels compared to ACT (p < 0.01 for both comparisons; Figure 1C). Given the unavailability of a reference genome for *T. sibiricus*, we performed *de novo* transcriptome assembly using Trinay with default settings to obtain transcripts and unigenes, followed by a quality control analysis of the assembled transcripts (Supplementary Figure S1A). The transcript sequence similarity was analyzed based on the Nr database, with *Ictidomys tridecemlineatus* showing the highest proportion at 35.8%, followed by *Homo sapiens* at 4.6%, *Rattus norvegicus* at 2.6%, *Bos mutus* at 2.4%, *Microcebus murinus* at 2.0%, and other species collectively accounting for 52.6% (Figure 1D). Additionally, gene function annotation was performed using the Swissprot reference database, which yielded 10 different species, with the top three in terms of unigene count being *Homo sapiens* (14,488 unigenes), *Mus musculus* (7,545 unigenes), and *Bos taurus* (3,000 unigenes) (Supplementary Figure S1B). With these transcriptome data assembled and annotated, we next applied PCA and heatmap analyses to identify the key group exhibiting significant variation across the different stages of hibernation.

### 3.2 Preliminary gene screening based on PCA/Heatmap analysis and quantity validation

PCA analysis revealed that among the five groups, ACT and E-EUT (both arousal states) clustered together, while M-DS and E-DS (both torpor states) formed another cluster. In contrast, M-EUT grouped separately, suggesting that the gene expression pattern in M-EUT is distinct from both the arousal and torpor states. This unique clustering indicates that M-EUT may be associated with maintaining specific hibernation-related functions (Figure 1E). The heatmap analysis further confirmed this finding. The rightmost three columns, representing the M-EUT group, displayed a distinct pattern of gene expression, with a clear downregulation in the lower portion of the heatmap and upregulation in the upper portion. In contrast, the left four groups exhibited a significantly different pattern of gene expression, particularly in the lower portion of the heatmap, where the M-EUT group showed a markedly different downregulation trend (Figure 1F). Subsequent upset plot analysis revealed a counter-intuitive phenomenon: while both ACT and M-EUT are arousal states, their gene expression profiles differed substantially, with 477 differentially expressed genes (DEGs) between ACT and M-EUT, compared to only 100 DEGs between ACT and M-DS (Figure 1G). Contrary to expectations, M-EUT, which during the euthermic phase, showed more DEGs than M-DS, a deep sleep phase. This unexpected result suggests that M-EUT might regulate a distinct set of genes crucial for re-entering torpor during hibernation. This phenomenon was not observed at the end of hibernation, where the DEGs between ACT and E-EUT were only 19, while ACT and E-DS had 254 DEGs. The difference between M-EUT and E-EUT lies in the former’s return to torpor, while the latter exits hibernation entirely and resumes a normal circadian rhythm. To explore this further, we conducted KEGG and GO enrichment analyses for the DEGs between ACT and M-EUT, separating upregulated and downregulated genes. The top 20 enriched pathways were all downregulated, with most related to neural regulation, such as Synaptic vesicle cycle, Neuroactive ligand-receptor interaction, Dopaminergic synapse, Potassium channel complex, Cation channel complex (Figure 1H; Supplementary Figure S1C). This pattern indicates that these downregulated genes in M-EUT may facilitate the transition from arousal back to the torpor state, essential for sustaining the recurring cycles of hibernation in *T. sibiricus*. We hypothesize that the genes differentially expressed in M-EUT may play a critical role in sustaining hibernation, particularly in facilitating the transition from arousal back to the torpor state, which is essential for the recurring cycles of hibernation in *T. sibiricus.*

### 3.3 Refined differential gene analysis with gene set filtering and pathway enrichment to identify key genes sustaining hibernation in *Tamias sibiricus*

In our experimental design, the comparison between ACT vs. M-EUT is critical, as it isolates the genes involved in the difference between a non-hibernating and a hibernating but aroused state. However, we recognize that not all of the genes in this comparison may be directly related to hibernation, as some may simply reflect differences in other physiological states between the two groups. To refine this comparison and filter out non-hibernation-related genes, we introduced the ACT vs. E-EUT comparison. Like ACT vs. M-EUT, both groups are in an aroused state, but E-EUT represents squirrels at the end of hibernation, preparing to exit the torpor-arousal cycle. By using ACT vs. E-EUT as a reference, we can eliminate genes related to general physiological states shared between these groups, leaving only those genes specific to sustaining hibernation. This filtering step ensures that the genes remaining after excluding ACT vs. E-EUT are more likely involved in the re-entry and maintenance of the torpor state. Meanwhile, the comparison between M-DS vs. M-EUT reveals the genes involved in transitioning between sleep and arousal states during hibernation. However, this comparison includes genes that reflect differences in wake–sleep states, and not all of them are specifically related to the maintenance of hibernation. To ensure that we specifically focus on hibernation-maintaining genes rather than genes associated with wake–sleep transitions, we included the E-DS vs. E-EUT comparison. This allowed us to filter out genes that are related to different wake–sleep states, leaving behind those specifically involved in the re-entry into the torpor state.

**Figure 2:**
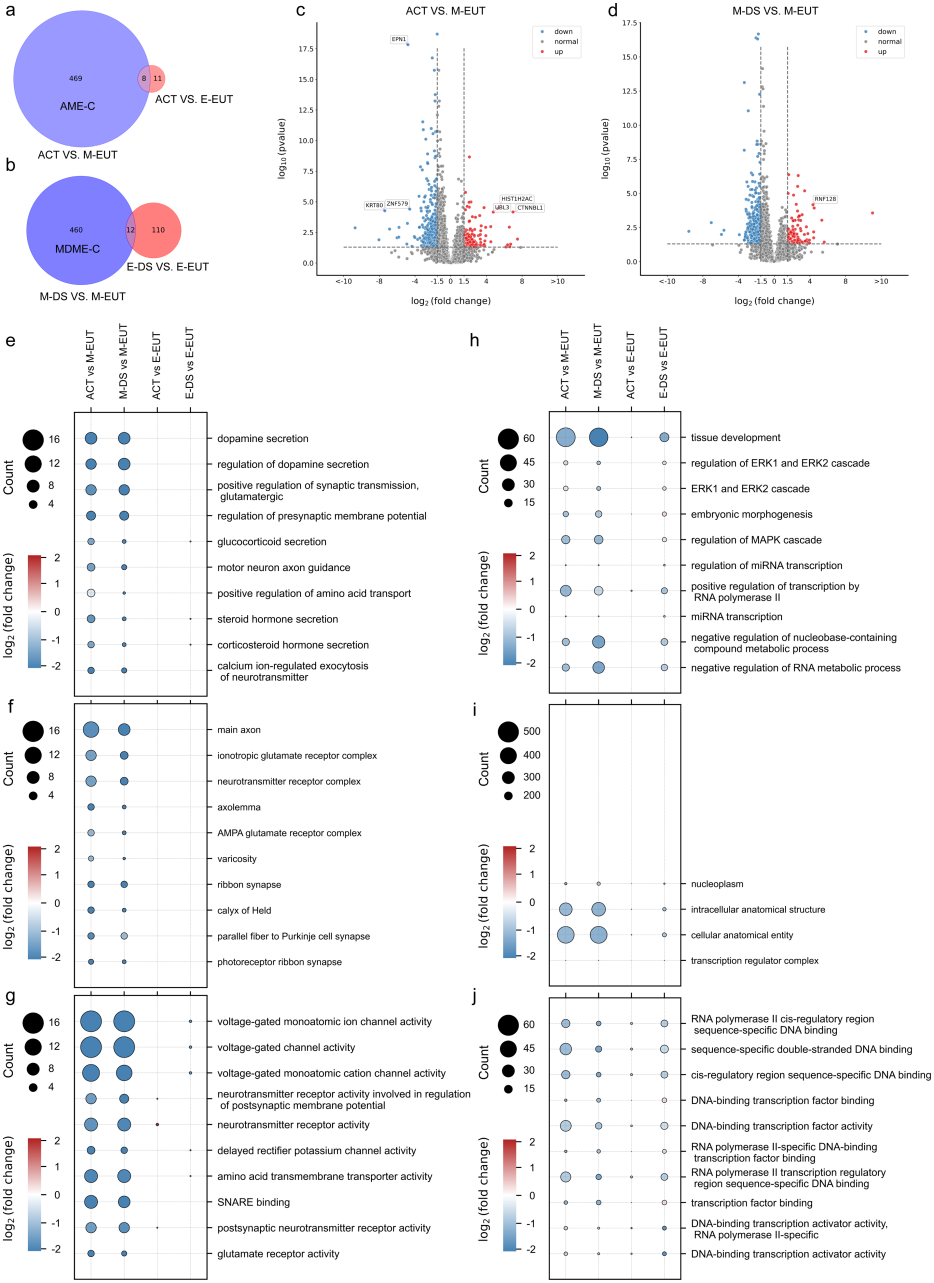
Comparative Venn diagrams, volcano plots, and Gene Ontology (GO) enrichment pathway charts between ACT, M-EUT, M-DS, E-EUT, and E-DE. **(A)** Venn diagram of transcriptomic genes differentially expressed between ACT vs. M-EUT and ACT vs. E-EUT. **(B)** Venn diagram of differentially expressed genes between M-DS vs. M-EUT and E-DS vs. E-EUT. **(C,D)** Volcano plot analysis of the differential gene expression between different comparisons, with blue indicating downregulation and red indicating upregulation. Adjusted *p*- value <0.05, |logFC| > 1.5. **(E–G)** GO enrichment pathway charts for ACT vs. M-EUT, ACT vs. E-EUT, M-DS vs. M-EUT, and E-DS vs. E-EUT, where circle size represents the number of genes in the pathway, and color indicates the magnitude of logFC. Each pathway chart was sorted by the enriched p-value from high to low in ACT vs. M-EUT, displaying the top 10 pathways with the lowest *p*-values. From top to bottom are Biological Process (BP) **(E)**; Cell Component (CC) **(F)**; Molecular Function (MF) **(G)**. **(H–J)** GO enrichment pathway charts for ACT vs. M-EUT, ACT vs. E-EUT, M-DS vs. M-EUT, and E-DS vs. E-EUT, where circle size represents the number of genes in the pathway, and color indicates the magnitude of logFC. Each pathway chart was sorted by the enriched p-value from high to low in E-DS vs. E-EUT, displaying the top 10 pathways with the lowest p-values. From top to bottom are BP **(H)**; CC **(I)**; MF **(J)**.

Building on our previous Venn diagram analysis and experimental logic, where we identified M-EUT as a key group in the overall comparison of the five conditions, we now performed a more focused Venn diagram analysis to refine our understanding of the differentially expressed genes in M-EUT. The first Venn diagram (Figure 2A) compares ACT vs. M-EUT with ACT vs. E-EUT, showing a significant number of unique differentially expressed genes in M-EUT, consistent with our earlier findings that M-EUT may play a crucial role in the maintenance of hibernation. Similarly, the second Venn diagram (Figure 2B) compares M-DS vs. M-EUT with E-DS vs. E-EUT, and both analyses further confirm the importance of M-EUT. These consistent results suggest that M-EUT contains important genes involved in the hibernation process, specifically those responsible for maintaining the torpor-arousal cycles. To refine our analysis and eliminate background noise, we applied background filtering to exclude genes shared with ACT vs. E-EUT and E-DS vs. E-EUT, as these groups do not contain the genes involved in maintaining hibernation. The filtered gene sets allowed us to focus on the unique genes in ACT vs. M-EUT and M-DS vs. M-EUT that are likely related to sustaining hibernation (Figures 2A,B). We further validated this approach by performing volcano plot analysis (Figures 2C,D), which displayed clear sets of upregulated and downregulated genes in ACT vs. M-EUT and M-DS vs. M-EUT, reinforcing the biological significance of these comparisons.

Following this analysis, we performed pathway enrichment, focusing first on the pathways enriched from ACT vs. M-EUT, and then examining how the same pathways were expressed in other groups, such as ACT vs. E-EUT, M-DS vs. M-EUT, and E-DS vs. E-EUT (Figures 2E–G). The consistent pathway enrichment between ACT vs. M-EUT and M-DS vs. M-EUT highlighted shared biological processes that are likely involved in sustaining hibernation, particularly those related to neurotransmitter secretion, ion channel activity, and transporter functions. These pathways support the notion that M-EUT plays a central role in maintaining the torpor-arousal cycles during hibernation. On the other hand, the right-side enrichment analysis (Figures 2H–J), which focused on pathways enriched from E-DS vs. E-EUT, revealed minimal overlap with the pathways enriched from ACT vs. M-EUT and M-DS vs. M-EUT. The pathways identified from E-DS vs. E-EUT were mostly related to general physiological functions, such as ERK1/2 signaling, mRNA transcription regulation, and MAPK pathways, rather than hibernation-specific processes. This lack of significant overlap further reinforces the idea that E-DS vs. E-EUT does not contain genes involved in the maintenance of hibernation, and thus acts as a negative control for our study. This brings us back to the complement sets, where AME-C (Complement of ACT vs. M-EUT with ACT vs. E-EUT) and MDME-C (Complement of M-DS vs. M-EUT with E-DS vs. E-EUT) represent purified gene sets that are likely involved in sustaining continuous hibernation. By removing the noise from the shared differentially expressed genes, we were able to focus on the key genes that regulate hibernation (Figures 2A,B). This two-step filtering process ensured that the gene sets we analyzed were specifically related to the maintenance of hibernation, as demonstrated by the pathway enrichment results.

### 3.4 Intersection and enrichment analysis of purified gene sets to identify hibernation-regulating pathways

**Figure 3:**
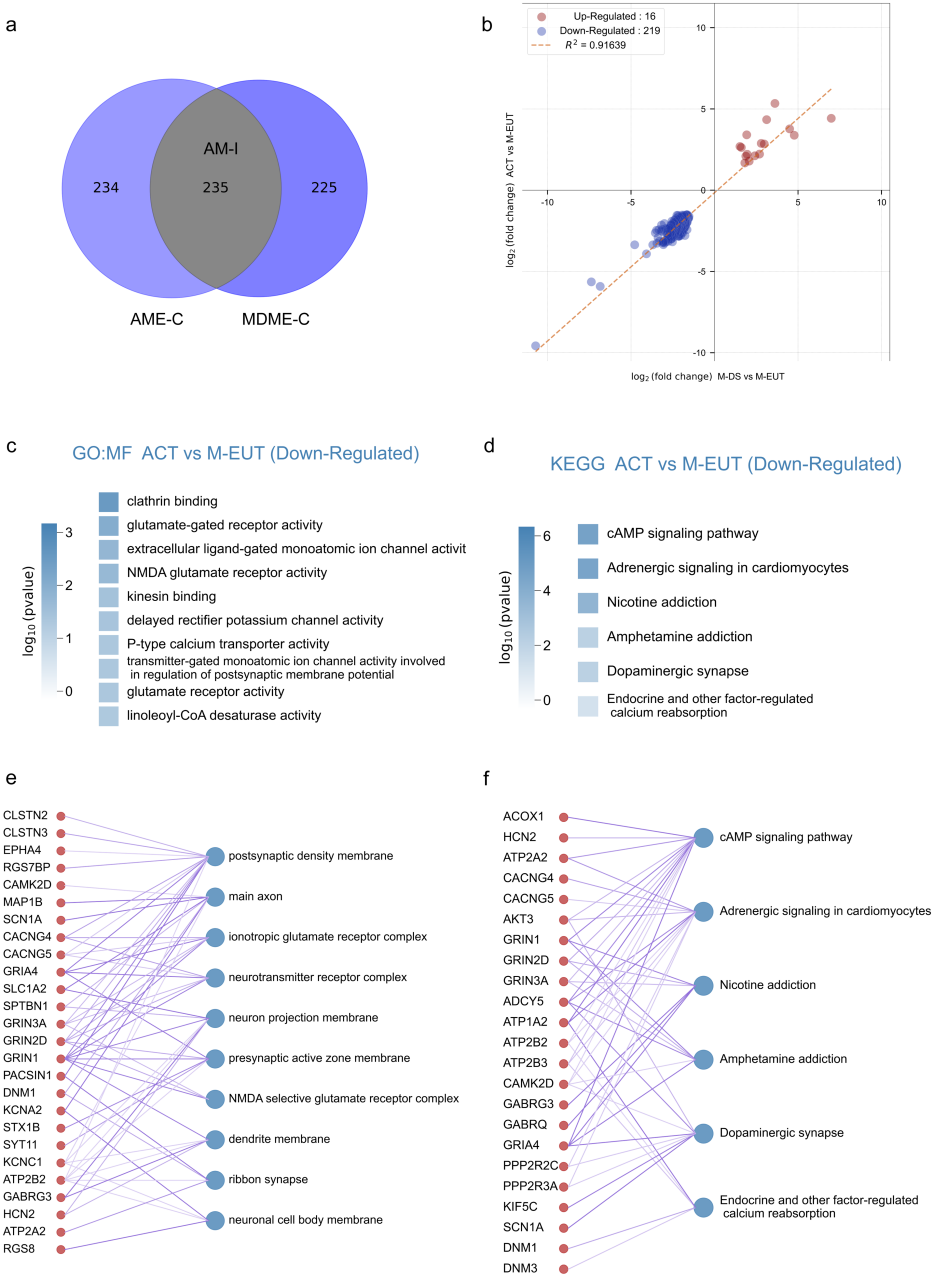
Intersection analysis and enrichment plots for the complementary sets of ACT vs. M-EUT and M-DS vs. M-EUT. **(A)** Venn diagram showing the intersection of the complementary sets of ACT vs. M-EUT and M-DS vs. M-EUT. **(B)** Linear regression analysis of the intersection of the complementary sets of ACT vs. M-EUT and M-DS vs. M-EUT. Points represent genes in the intersection; red indicates upregulation, and blue indicates downregulation. R^2^ represents the coefficient of determination, where values closer to 1 indicate a higher correlation. **(C,D)** Gene Ontology - Molecular Function (GO-MF) enrichment pathway chart and KEGG enrichment pathway chart for the genes in the intersection of the complementary sets of ACT vs. M-EUT and M-DS vs. M-EUT, respectively. Color intensity represents log_10_ (p-value), with darker colors indicating larger log_10_ (p-value) magnitudes. **(E,F)** Connectivity plots between genes at the intersection of the complementary sets of ACT vs. M-EUT and M-DS vs. M-EUT, and their enriched GO-MF pathways **(E)** and KEGG pathways **(F)**. The left side shows genes from the intersection of ACT vs. M-EUT and M-DS vs. M-EUT complementary sets, and the right side shows the enriched GO-MF pathways **(E)** and KEGG pathways **(F)**.

Although the differentially expressed genes contained in AME-C and MDME-C were already purified through gene set filtering, there is still a possibility of false positives. To further reduce these false positives, we took the intersection of the two sets, AME-C and MDME-C, to identify the most likely candidate genes involved in maintaining hibernation. This intersection, denoted as AM-I, yielded 235 differentially expressed genes (Figure 3A), thereby further refining the data to focus on the most relevant genes. Correlation analysis was performed on these 235 genes (Figure 3B), revealing that there were fewer upregulated genes (Merriman et al., 2012) which did not enrich any specific pathways, whereas the majority were downregulated genes (219). Subsequent GO and KEGG enrichment analyses showed that these downregulated genes were significantly enriched in pathways related to ion channels, transporter proteins, neurotransmitters, and signal transduction (Figures 3C,D; Supplementary Figure S2). This further supports the hypothesis that these pathways play a crucial role in maintaining the torpor-arousal cycles during hibernation. In the GO-MF enrichment analysis, similar to previous results (Figures 2E–J), pathways related to ion channels and neurotransmitters such as voltage-gated monoatomic ion channel activity, voltage-gated monoatomic cation channel activity, voltage-gated monoatomic ion channel activity involved in the regulation of presynaptic membrane potential, ionotropic glutamate receptor activity, neurotransmitter binding, NMDA glutamate receptor activity, voltage-gated sodium channel activity were enriched. In the KEGG enrichment analysis, the cAMP signaling pathway ranked first, potentially acting as a second messenger downstream of ion channels in the GO-MF. Additionally, Endocrine and other factor-regulated calcium reabsorption was also an enriched pathway in the KEGG analysis.

For a more detailed and intuitive analysis of each pathway, we displayed the genes contained in each pathway (Figures 3E,F), where the thickness of the lines connecting the genes and pathways reflects the significance (thicker lines indicate stronger significance). First, we observed many genes from the same family that appeared in the results of GO-MF and KEGG enrichment analyses, working together. For instance, voltage-gated potassium (Kv) channels-related genes, such as Kcna6, Kcnc1, and Kcng4, and ATPase family genes, such as Atp1a2, Atp2a2, Atp2b2, and Atp2b3, were enriched. Moreover, pathways enriched with clathrin binding, kinesin binding, and others not directly associated with ion channels, transporter proteins, or neurotransmitter genes are also involved in related functions. For example, the protein encoded by Epn1 in the clathrin binding pathway participates in regulating receptor-mediated endocytosis (Nakashima et al., 1999); Neurocalcin Delta (NCALD) encodes a member of the calcium-binding protein neuronal calcium sensor (NCS) family, which undergoes conformational changes when intracellular calcium levels rise, participating in vesicle-mediated transport functions (Ivings et al., 2002); Syt9, Syt11, Syt12, and Syt13 are all members of the synaptotagmin gene family and mediate calcium-dependent regulation of membrane trafficking during synaptic transmission.

To summarize, by taking the intersection, we obtained an AM-I gene set with a lower false-positive rate. Through GO-MF and KEGG enrichment analysis, we obtained more focused ion channel, and transporter enrichment pathways, which may be involved in the regulation of hibernation during the hibernation process of *T. sibiricus.*

### 3.5 Annotated transcriptome analysis reveals Ion Channel enrichment and alternative splicing events in *Tamias sibiricus* hibernation

**Figure 4:**
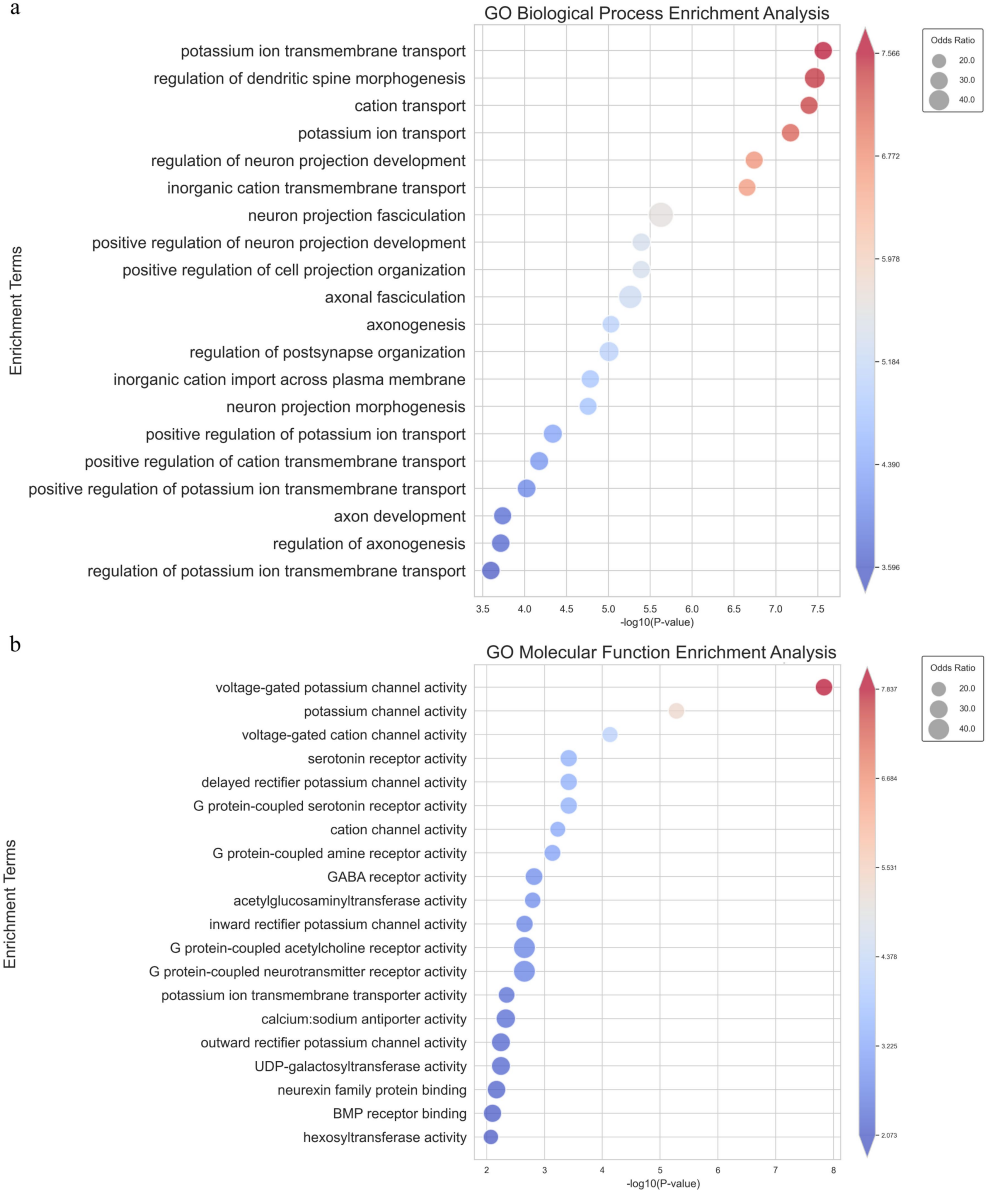
GO_BP and GO_MF enrichment pathway analysis of intersecting genes from the parameterized transcriptome analysis. **(A)** Bubble chart showing the top 20 significantly enriched pathways in GO Biological Process, where the size of each bubble represents the odds ratio and the color reflects the-log10 (*p*-value). **(B)** Bubble chart showing the top 20 significantly enriched pathways in GO Molecular Function, with similar representation where bubble size corresponds to the odds ratio and color represents-log10 (*p*- value).

To further validate and complement our previous results obtained from the non-annotated transcriptome analysis, we conducted an annotated transcriptome analysis by aligning the detected genes from the *T. sibiricus* genome with our RNA-sequencing data (Supplementary Table S2). Following the same gene set filtering and intersection procedures as before, we focused on the most relevant genes and pathways for maintaining hibernation. Similar to the non-annotated analysis, we observed significant enrichment in ion channels and transporter-related pathways, particularly those associated with sodium and potassium ion channels. Notably, in the top 20 enriched pathways from the GO-BP analysis, five were related to ion channels, with potassium ion transmembrane transport ranked first and potassium ion transport ranked fourth (Figure 4A). In the GO-MF analysis, the top two most significantly enriched pathways were all related to ion channels, with the top three pathways specifically associated with ion channel activity (Figure 4B). These pathways include voltage-gated potassium channel activity, potassium channel activity, and voltage-gated cation channel activity. In addition, consistent results were observed in both the GO-CC and KEGG enrichment analyses (Supplementary Figures S4A,B). This further supports our hypothesis regarding the role of ion channels in the hibernation process. This enriched data aligns with our earlier non-annotated analysis, strengthening the hypothesis that ion channels—particularly those related to potassium, calcium, and sodium ions—are crucial for maintaining the periodic torpor and arousal cycles during hibernation.

In the course of our annotated transcriptome analysis, we identified 19 alternative splicing events among the genes in the final gene set intersection (Supplementary Table S3). Notably, several of these splicing events were associated with genes involved in neural regulation. Among them, SLC24A2, Sytl5, and FAAH were found to exhibit significant alternative splicing. SLC24A2 and Sytl5 showed exon skipping (SE), whereas FAAH demonstrated mutually exclusive exons (MXE). These splicing variants suggest that alternative splicing may play a role in modulating the function of these genes in the context of neural regulation, possibly influencing processes that are crucial for maintaining the hibernation state.

### 3.6 Validation of key hibernation-related genes via qRT-PCR analysis

**Figure 5:**
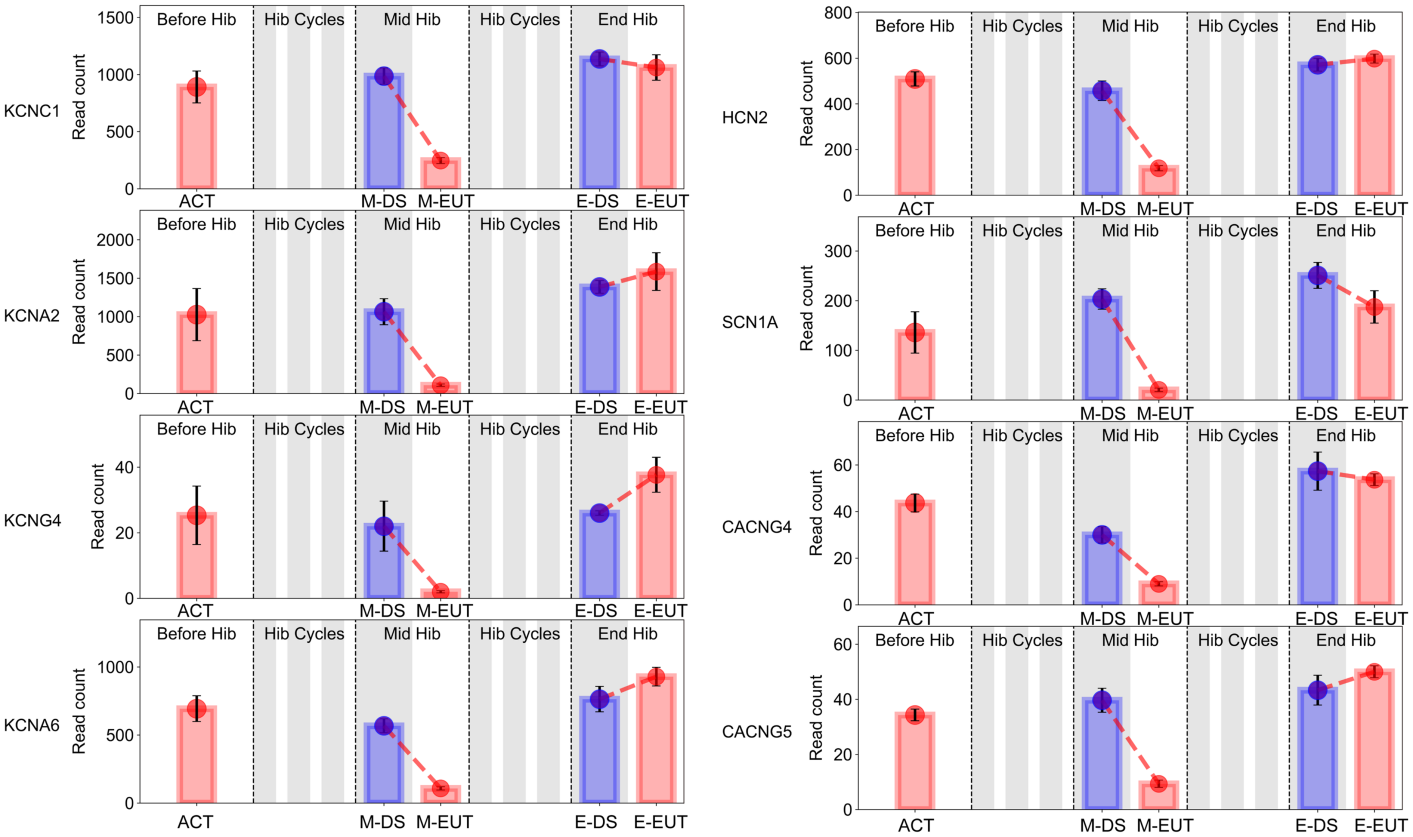
Bar graphs of sodium (Na+), potassium (K+), and calcium (Ca2+) gene expression in the hypothalamus of *T. sibiricus* at different time points. Each graph, from left to right, represents the following stages: pre-hibernation (Before Hib), hibernation cycles (Hib Cycles), mid-hibernation (Mid Hib), and end-of-hibernation (End Hib). The color of the sample represents the sleep/euthermia phase: red for the euthermia phase and blue for the deep sleep phase. Time points are ordered from left to right as ACT, M-DS, M-EUT, E-DS, and E-EUT.

In the GO-MF and KEGG enrichment analyses, we discovered that these genes from the same family were enriched in different pathways (Figures 3E,F), acting synergistically. Therefore, we organized the expression levels of genes from the same family or with similar functions and presented their changing trends during hibernation bouts (Figure 5; Supplementary Figure S3). The figure shows the common characteristics of the changing trends in these genes during hibernation bouts. In End Hib, the changes in gene expression between E-DS and E-EUT were minimal or unchanged. However, in Mid Hib, the gene expression levels were higher in M-DS, where the body temperature was lower and in a torpor state, whereas the expression levels decreased in M-EUT when the body temperature rose and was in an arousal state. Additionally, the genes in Before Hib did not show significant changes, and there were no significant differences compared to the two groups in End Hib. That is, these genes only showed a significant decline during M-EUT in Mid Hib. This result strongly supports our hypothesis that the downregulation of these genes plays a role in maintaining hibernation in *T. sibiricus*. Specifically, this includes genes related to the excitatory neurotransmitter glutamate, such as the ionotropic glutamate receptor subunits Grin1, Grin2d, Grin3a, and Grin4 (Grins); potassium voltage-gated channel genes involved in regulating intracellular and extracellular sodium and potassium ion concentrations, such as Kcnc1, Kcna2, Kcng4, Kcna6 (Kcns); Sodium Leak Channel gene Nalcn, Sodium Voltage-Gated Channel genes Scn1a; Hyperpolarization-Activated Cyclic Nucleotide-Gated gene Hcn2; calcium ion regulation-related calcium voltage-gated channel genes Cacng4, Cacng5 (Cacngs), and calcium ion-mediated synaptotagmin genes Syt9, Syt11, Syt12, Syt13 (Syts); and sodium-potassium ATPase genes Atp1a2, Atp2a2, Atp2b2, Atp2b3 (Atps). These genes may serve as key players in maintaining the continuation of hibernation in *T. sibiricus*.

**Figure 6:**
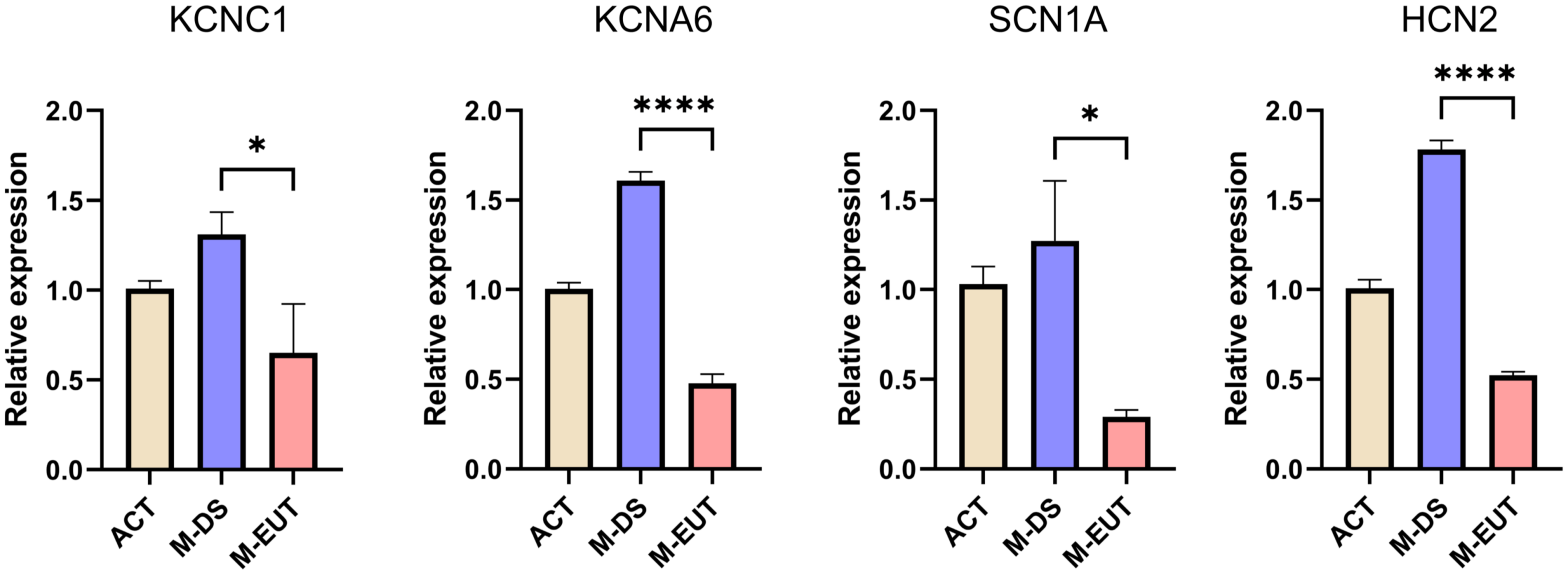
Relative expression levels of four genes (KCNC1, KCNA6, SCN1A, and HCN2) in three groups. ACT, M-DS, and M-EUT. Gene expression levels were measured by qRT-PCR and normalized against control genes. The error bars represent the standard deviation of three biological replicates. Significant differences between groups were determined by statistical analysis (*p < 0.05, ****p < 0.0001), indicating downregulation of these genes in the M-EUT group compared to the M-DS group.

In the final step of our analysis, we validated the expression of four key ion channel genes—KCNC1, KCNA6, SCN1A, and HCN2—using qRT-PCR (Figure 6). The qRT-PCR results confirmed the differential expression observed in the transcriptomic analysis, particularly during the M-EUT phase, where these genes exhibited significant downregulation compared to the M-DS phase. KCNA6 and HCN2 showed highly significant differences (****p < 0.0001), while KCNC1 and SCN1A also demonstrated significant downregulation (*p < 0.05). These findings corroborate the earlier transcriptomic results and provide strong evidence that these genes are involved in regulating ion balance and neural excitability during the hibernation cycle. Specifically, the downregulation of these genes in the M-EUT phase likely supports the re-entry into the torpor state and sustains the continuous cycles of hibernation. By suppressing neural excitability and controlling ion flow, these genes may facilitate the transition back into torpor, helping *T. sibiricus* maintain its hibernation cycle during the hibernation. Thus, the qRT-PCR validation highlights the importance of these four key genes in sustaining hibernation in *T. sibiricus*. Their downregulation is closely associated with the regulation of ionic balance and neurotransmission during hibernation bouts, reinforcing their potential role in the maintenance of hibernation.

In summary, through the analysis of transcriptomic data from the ACT, M-DS, M-EUT, E-DS, and E-EUT in *T. sibiricus*, we identified several genes and pathways that are functional in maintaining the continuation of hibernation. Among these, the ion channel genes KCNC1, KCNA6, SCN1A, and HCN2 were validated through qRT-PCR analysis. KCNA6 and HCN2, in particular, showed highly significant downregulation during the euthermia phase, suggesting their crucial role in facilitating the re-entry into torpor and sustaining continuous hibernation cycles. These findings provide valuable insights into the molecular mechanisms underlying hibernation, with a focus on ion channel regulation and neurotransmitter balance.

## 4 Discussion

In our study, we faced a critical decision regarding the choice between reference-based and *de novo* transcriptome analysis methods. Since *T. sibiricus* is a non-model organism, its genomic annotations are not as comprehensive as those available for model species such as humans or mice. As a result, reference-based analysis, although more accurate and with a lower error rate, might overlook key genomic information, potentially leading to a higher false negative rate. On the other hand, *de novo* transcriptome analysis, which does not rely on a reference genome, allows for a more comprehensive exploration of gene expression, but it comes with an increased risk of false positives due to the absence of genome-specific annotations. Given these challenges, we initially opted for a *de novo* analysis to explore the mechanisms underlying hibernation in *T. sibiricus*, as it provided a broader scope of potentially relevant genes and pathways that might not be captured by a reference-based approach. This strategy allowed us to identify a wide range of differentially expressed genes. Following this, we integrated genomic data from our newly sequenced and annotated *T. sibiricus* genome to cross-validate and refine our initial findings. This approach helped ensure that our conclusions were not only more comprehensive but also validated against a higher-quality reference, thereby improving the accuracy of our results. Additionally, we employed RT-qPCR to further confirm the expression patterns of selected genes, providing an independent validation of our transcriptomic findings. In summary, our study benefited from the complementary use of both *de novo* and reference-based analyses. This dual approach enabled us to capture a more holistic view of the genetic mechanisms involved in hibernation while minimizing the risks of both false positives and false negatives.

The present study aimed to identify the mechanisms underlying hibernation maintenance in *T. sibiricus* by sampling the hypothalamus at multiple time points throughout the hibernation bout and performing transcriptomic data analysis. We found enriched pathways and differential gene expression changes in the hypothalamus during the M-EUT phase of hibernation, which facilitated re-entry into sleep and sustained the hibernation state. Specifically, pathways associated with sodium and potassium ion channels as well as the Kcns, Scns, Hcns, and Nalcn genes were significantly downregulated. This process likely inhibits the generation of action potentials in neurons, thereby maintaining hibernation. Changes in ion channels during hibernation have been observed in various species, though most research has focused on adaptations in peripheral tissues to conditions such as low temperature or hypoxia (Offstad et al., 1994; Yang et al., 2021; Maltsev, 2018; Eleftheriadis et al., 2018; Gillen et al., 2023). Some studies have also explored the role of peripheral organs, like the skin and retina, in sensing the hibernation environment (Nagai et al., 2012; Mehta et al., 2013). However, research on ion channels within the central nervous system (CNS) remains limited. Among the existing studies, CNGA3 and TRP channels have been implicated in temperature sensing and thermoregulation, highlighting their potential role in hibernating animals (Feketa et al., 2020; Li et al., 2022). Our study provides the first insights into the dynamic transcriptome of the hypothalamus across different time points in *T. sibiricus* during hibernation, uncovering the potential role of sodium and potassium ion channel pathways. Specifically, genes within the Kcns, Scns, Hcns, and Nalcn families appear to play a crucial role in sustaining hibernation. These hypothalamic ion channels may complement the roles of other types of ion channels, such as CNGA3 and TRP channels, which are involved in different aspects of hibernation.

Currently, the animals frequently used as hibernation models include arctic ground squirrels, thirteen-lined ground squirrels, Japanese chipmunks, Siberian hamsters, and Marmota. One of the most widely used hibernation animal models is the thirteen-lined ground squirrel (*Ictidomys tridecemlineatus*) (Gillen et al., 2023; Feketa et al., 2020; Regan et al., 2022; Dai Pra et al., 2022; Feng et al., 2019; Ou et al., 2018; Fu et al., 2020; Gillen et al., 2021). The thirteen-lined ground squirrel is a compulsory hibernator with a long hibernation duration that can last up to 18 days. With a low body temperature and suppressed metabolism, it can survive for more than 6 months in underground burrows or laboratory hibernation conditions without food and water (Andrews, 2019). Viktor V. Feketa et al. studied the cold sensitivity mechanism of the hypothalamic preoptic area (POA) in thirteen-lined ground squirrels and discovered that the cyclic nucleotide-gated ion channel CNGA3 could serve as a molecular marker for the neuronal circuitry underlying temperature regulation in the hypothalamus (Feketa et al., 2020). Additionally, Neel S. Singhal, Sarah A. Rice, and others primarily use Arctic Ground Squirrels (AGS) for hibernation research (Rice et al., 2020; Singhal et al., 2020). Due to AGS’s ability to survive harsh winter environments with strong metabolic suppression and core temperature reduction capabilities, they are referred to as ‘extreme’ hibernators and thus serve as excellent models for studying the repair capacity of reperfusion injury and intrinsic tolerance to metabolic stress or damage (Singhal et al., 2020; Barnes, 1989). Gabriela M. Pinho, Liang Bai, and others mainly use Marmota (including the Himalayan marmot (*Marmota himalayana*) and yellow-bellied marmots (*Marmota flaviventer*)) for hibernation research (Pinho et al., 2022; Bai et al., 2019). Marmota possess unique biological characteristics, such as hibernation, deep burrowing, thick fur, and increased body size, which may be related to their evolutionary response to adverse environmental pressures (Cardini and O’Higgins, 2005). Moreover, due to their long hibernation duration (7–8 months per year) and longevity, Gabriela M. Pinho has validated the ‘hibernation–aging hypothesis’ from an epigenetic perspective (Pinho et al., 2022; Armitage et al., 2003). Besides these, some niche animal models have also made significant contributions to the study of hibernation mechanisms. For instance, Riyue Bao et al. elucidated the mechanism related to seasonal energy balance by thyroid hormone T3 in the hypothalamus of Siberian hamsters (*Phodopus sungorus*) through genomic and transcriptomic sequencing analysis (Bao et al., 2019). Daisuke Tsukamoto et al. investigated the energy metabolism changes in the liver during *T. sibiricus* hibernation and the transcriptional regulation of related protein genes (Tsukamoto et al., 2019; Tsukamoto et al., 2017; Takamatsu et al., 1993; Kojima et al., 2000). In our experiment, we selected the *T. sibiricus* as the experimental animal to investigate the mechanisms of hibernation. The *T. sibiricus* belongs to the subfamily Xerinae, within the family Sciuridae of the order Rodentia. This hibernation animal model offers advantages, such as shorter bouts of hibernation, ease of acquisition, and small size. It is nocturnal, with wild populations naturally distributed in Russia and some East Asian countries (China, Mongolia, South Korea, and Japan). Furthermore, compared to hoarding animals, *T. sibiricus*, being food-caching animals that wake up every 3–5 days to replenish their food supply, aligns more closely with human eating habits. This makes them particularly suitable for simulations in astronauts or other humans without exposing individuals to prolonged severe energy deficiency, thereby reducing the risks associated with human hibernation simulations. Ran Li et al. conducted whole-genome sequencing on *T. sibiricus*, providing a reference for genomic experimental data and basic biological characteristics of the species (Li et al., 2022). Therefore, various hibernating mammals, including *T. sibiricus*, provide suitable animal models for human investigations of hibernation, reperfusion injury, and thermoregulation mechanisms.

Over time, many brain regions within the central nervous system, including the cortex, hippocampus, medulla, and forebrain, have been recognized as closely associated with hibernation processes (Cerri et al., 2013; Fu et al., 2020; Bhowmick et al., 2017; Yamada et al., 2019; de Veij Mestdagh et al., 2024). For example, a study in 2020 revealed dynamic RNA regulation across three major brain regions in the thirteen-lined ground squirrel—the forebrain, hypothalamus, and medulla—throughout hibernation. This study highlighted changes in RNA stability and transcriptional regulation under varying temperatures and metabolic suppression (Fu et al., 2020). Additionally, selectively inhibiting neurons in the rostral ventromedial medulla (RVMM) was found to induce a hibernation-like suspended animation state, suggesting that this region might play a critical role in facilitating hibernation-associated metabolic downregulation (Cerri et al., 2013). In the cortex, researchers have observed dynamic changes in tau hyperphosphorylation and dephosphorylation during hibernation. These fluctuations in tau phosphorylation status indicate a reversible adaptation, possibly as a neuroprotective response to the extreme conditions of hibernation (Yamada et al., 2019). Another study discovered that the AMPK-eEF2 signaling pathway in the cortex of hibernating animals is crucial for adapting to reduced metabolic demands. This pathway helps explain how the brain conserves energy during hibernation by modulating protein synthesis and metabolic rates in response to extreme environmental conditions (de Veij Mestdagh et al., 2024). While numerous brain regions are closely tied to hibernation processes, the hypothalamus, as a critical center for regulating energy metabolism, feeding behavior, and sleep in mammals, remains central to understanding the underlying mechanisms of hibernation. This region has been extensively studied due to its significant role in balancing these physiological processes, which are essential for sustaining hibernation (Chen et al., 2018; Harrold et al., 1998; Bao et al., 2019).

The hypothalamus is considered a key center for regulating hibernation in mammals (Mohr et al., 2020; Andrews, 2019; Drew et al., 2007; Frare et al., 2018; Frare et al., 2021; Junkins et al., 2022; Helwig et al., 2009). Studies have shown that many neurotransmitter receptors (such as opioid receptor antagonists and GABAergic receptors) play a role in maintaining hibernation. Intracerebroventricular injection of naloxone (a non-selective opioid receptor antagonist) or naloxonazine (a selective *μ*1-opioid receptor antagonist) can arouse Syrian hamsters from hibernation (Tamura et al., 2005). During the maintenance phase of hibernation, *β*-endorphin synthesized in ARC neurons regulates Tb by activating μ-opioid receptors in PO, AH, VMH, DMH, and PH (Tamura et al., 2012). Different αβγ subunits of GABA receptors also play important roles in the induction of hibernation onset or arousal states in Syrian golden hamsters (Alò et al., 2010). The activation of A1AR in the central nervous system can promote metabolic suppression during torpor episodes with the help of seasonal shifts in purinergic signal sensitivity (Jinka et al., 2011). Somayeh Mousavi et al. revealed seasonal changes in neuropeptides and peptide hormones in the hypothalamus and pituitary of hibernating mammals through peptidomics analysis (Mousavi et al., 2023). Additionally, Tohru M. Takahashi et al. also discovered that non-hibernating mammals could be induced into hibernation by regulating neural circuits in the hypothalamus (Takahashi et al., 2020). Similarly, in our study, we found significant downregulation of pathways involving metal ion channels or transporters in the hypothalamus, as well as significant downregulation of glutamate receptor genes, indicating unique changes in the hypothalamus during hibernation, which may play an important role in maintaining the continuation of hibernation.

In the results of AM-I, pathways involving metal ion channels and transporters were significantly enriched. However, research demonstrating the direct regulation of hibernation by metal ion channels and transporters is limited. Studies have shown that rhythmic changes in biological rhythms, core body temperature, and basal metabolism are major characteristics of hibernation, which are also accompanied by changes in signs such as decreased blood pressure, heart rate, and respiratory depression (Mohr et al., 2020; Xie et al., 2022; Körtner and Geiser, 2000; Carey et al., 2003; Geiser, 2004; Krystal et al., 2013; Heller and Ruby, 2004). Certain specific ion channels or metal ions and transporters in the hypothalamus regulate mammalian biological rhythms, body temperature, respiration, metabolism, blood pressure, and other signs (Reimúndez et al., 2023). The ion channel TRPM8 acts as a thermosensor involved in the regulation of central and peripheral clocks and the circadian rhythm control of Tc (Reimúndez et al., 2023; Voronova et al., 2022). Activating TRPM8 in rat skin can induce changes in gene expression in the hypothalamus and induce changes in thermoregulatory responses (Voronova et al., 2022). The regulatory role of hypothalamic ion channels in blood pressure has also been confirmed (Geraldes et al., 2018), and evidence supports that changes in hypothalamic neuronal activity, through changes in ion channels or intrinsic membrane properties, can excite or inhibit the sympathetic nervous system, thereby regulating blood pressure changes (Geraldes et al., 2014; Sonner et al., 2011; Geraldes et al., 2016; Li et al., 2014). Ion channels and receptors in the paraventricular nucleus (PVN) of the hypothalamus play an important role in regulating neuronal activity and endocrine functions, which are related to the regulation of energy balance and glucose metabolism. Knockout of ASIC1a led to significant weight gain, glucose intolerance, and insulin resistance (Wang et al., 2022). Additionally, during local acidosis, orexin neurons in the lateral hypothalamus can exert excitatory effects on respiration through the ASIC1 ion channel, indicating that hypothalamic ion channels can also regulate respiration to some extent. Furthermore, leptin can activate the sodium leak channel (NALCN), thereby depolarizing glutamatergic (VGluT2) neurons, increasing energy expenditure, and simultaneously increasing respiration (Do et al., 2020; Song et al., 2012). The electrophysiological activity of neurons in the hypothalamus depends on the activity of different types of voltage-and ligand-gated ion channels and transporters (Kline, 2017; Zhang et al., 2017; Simms and Zamponi, 2014). Therefore, the electrophysiological activity of neurons in the hypothalamus may also reflect, to some extent, the impact of ion channels and transporters on hibernation-related signs. Neurons in the suprachiasmatic nucleus of the hypothalamus exhibit circadian oscillations in spontaneous repetitive discharges, driving daily rhythms in physiology and behavior (Reppert and Weaver, 2001; Hastings et al., 2018; Michel and Meijer, 2020). One possible mechanism for the hypothalamic regulation of heart rate is the direct influence of the central biological clock of the suprachiasmatic nucleus on cardiac electrophysiology through various neurohumoral factors, particularly the autonomous nervous system (Tong et al., 2013; Schroeder et al., 2011; Sei et al., 2008; Curtis et al., 2007; Black et al., 2019). Increased excitability of the hypothalamus contributes to the elevation of circulating vasopressin levels and sympathetic nervous system activity (Carmichael and Wainford, 2015). Therefore, ion channels might sustain hibernation in *T. sibiricus* by directly regulating its biological rhythms, basal metabolic rate, heart rate, and respiration, or by affecting the hibernation of *T. sibiricus* activities through modulating the electrophysiological activity of hypothalamic neurons. In summary, ion channels may serve as a potential mechanism underlying hibernation maintenance in the hypothalamus of *T. sibiricus*.

Glutamate is the most abundant free amino acid in the brain and is a major excitatory neurotransmitter. As an amino acid derived from glucose, glutamate participates in nitrogen balance through the glutamine cycle and mediates its effects through NMDA, AMPA, kainate, and metabotropic receptors. Previous studies have explored the role of glutamate during hibernation in rodents. Inhibition of NMDA-type glutamate receptors induces arousal in hibernating arctic ground squirrels; however, the wakefulness effect after NMDAR activation only exists peripherally or around the ventricles, and central administration does not induce awakening in AGS (Jinka et al., 2012; Daikhin and Yudkoff, 2000). However, this study still illustrates how metabolic flux affects the neural mediation of intermittent arousal/sleep states. Metabolic flux influences glutamatergic signaling through its effect on substrate availability and regulation by metabolic end products. Since glutamate is extracted from glucose via the pyruvate carboxylase pathway. Recycling glutamate from glutamine through the glutamine cycle also maintains the supply of glutamate unless excess nitrogen drives glutamine equilibrium (Hamberger et al., 1979; Oz et al., 2004). Therefore, the two sources of glutamate, namely glucose and the glucose-glutamine cycle, may be restricted during prolonged lethargy. Low glucose levels reduce the supply of glutamate, limiting the capacity of the glucose-glutamine cycle. The reduction in glucose is one of the most potent biomarkers of glutamate inhibition (Serkova et al., 2007; Epperson et al., 2011). In our blood glucose testing and transcriptome sequencing analysis results, the blood glucose concentration of *T. sibiricus* remained declining throughout hibernation (Figure 1C). Additionally, in non-natural hibernating mammals, a group of glutamatergic Adcyap1-positive cells was discovered in the hypothalamus, the activity of which precisely determines when mice naturally initiate and exit torpor, and inhibition disrupts the natural process of entry, maintenance, and arousal from torpor (Hrvatin et al., 2020). This suggests that glutamate receptors may have a unique role in hibernation.

Our findings suggest that during the euthermia phase of hibernation in *T. sibiricus*, the expression of genes associated with sodium and potassium ion channels in the hypothalamus was downregulated. This potentially indicates inhibition of neuronal impulses in the hypothalamus during this period, which may facilitate the re-entry of the *T. sibiricus* into the sleep state and sustain the ongoing hibernation bout. Notably, sodium and potassium ion channels were significantly enriched in both GO and KEGG enrichment analyses. Within these enriched pathways, gene families such as Kcn, Scn, and Hcn exhibited consistent trends, all being downregulated during the euthermia phase of hibernation. These pathways and genes might play a role in maintaining hibernation in the *T. sibiricus*. We have uncovered potential differential gene expression specific to M-EUT, which may be unique to the maintenance of the hibernation process in *T. sibiricus*. The expression of these genes allows *T. sibiricus* to awaken from torpor to re-enter the sleep state. Our pioneering focus on the mechanisms involved in maintaining Mid Hib provides new perspectives for human research on the hibernation mechanisms of mammals.

## Data Availability

We acknowledge that the transcriptomic datasets referenced in the manuscript are available in the NCBI Sequence Read Archive (SRA) repository, BioProject PRJNA1101579, as stated. However, the genomic data referenced in this study is still under review and therefore not yet publicly available. The project ID for the genomic data is PRJNA1192718, and it will be accessible through the NCBI repository once the review process is complete.
