# Supplementary material for: Multiple time points of transcriptome analysis revealed altered genes involved in maintaining hibernation in the hypothalamus of Tamias sibiricus
Source: Front Neurosci. 2024 Dec 23;18:1501223. doi: 10.3389/fnins.2024.1501223 (PMC11701215; doi:10.3389/fnins.2024.1501223)
Supplement: Supplementary file 1 [file Data_Sheet_1.pdf]

Multiple time points of transcriptome analysis revealed altered genes involved in maintaining hibernation in the hypothalamus of *Tamias sibiricus*

Tian Zhang[1,2†], Chao Yang[2†*], Yaxiu Guo[2†], Zihan Xu[3], Minbo Zhao[4], Feng Wu[2], Hongyu Zhang[2], Hailong Wang[2], Xiukun Sui[2], Siyu Jiang[3] , Rongqiao He[5], Zhongquan Dai[2], Ying Liu[1*], Yinghui Li[2,3*]

[1] (School of Life Sciences, Beijing University of Chinese Medicine, Beijing, China, 102401)

[2] (National Key Laboratory of Space Medicine, China Astronaut Research and Training Center, Beijing, China, 100094)

[3] (School of Life Science and Technology, Harbin Institute of Technology, Harbin, China, 150001)

[4] (Department of Pathology and Forensics, Dalian Medical University, Beijing, China, 116044)

[5] (State Key Laboratory of Brain and Cognitive Science, Institute of Biophysics, Chinese Academy of Sciences, Beijing, 100101, China)

† These authors contributed equally to this work and shared the first authorship

* Correspondence:

Ying Liu, Yinghui Li, Chao Yang

yingliu@bucm.edu.cn, yinghuidd@vip.sina.com, zhangxiuyc@sina.com.

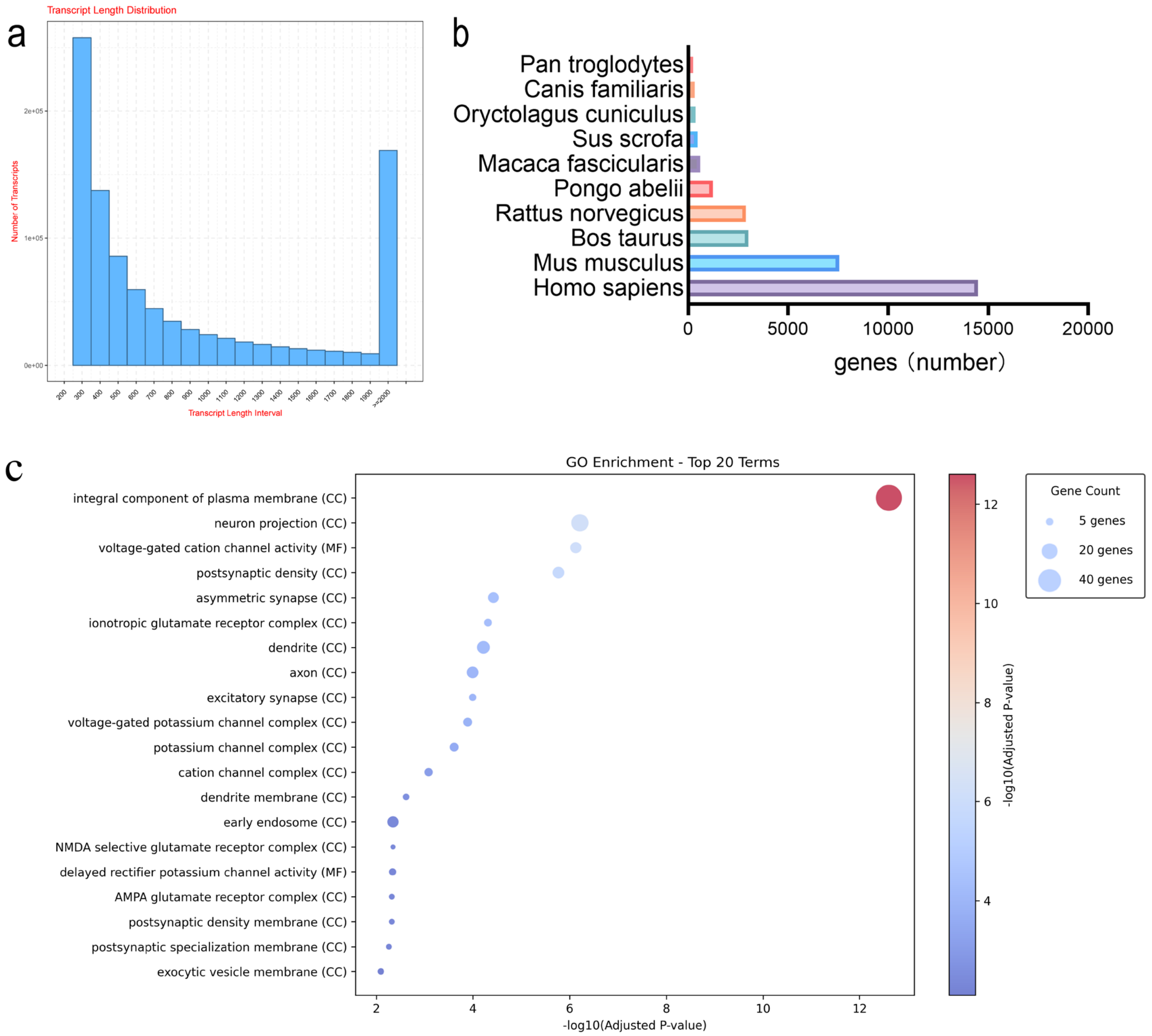

Figure S1. Quality control chart for transcriptomics de novo sequencing and species match chart of gene annotations from the Swissprot database. **a** Transcript length distribution. The x-axis represents the number of transcripts, and the y-axis represents the transcript length interval. **b** Gene function annotation results based on the Swissprot reference database. **c** Top 20 GO pathways enriched for downregulated differentially expressed genes (DEGs) between the ACT and M-EUT groups. Pathways are ranked by adjusted p-value. The x-axis represents the enrichment score, calculated as -log10(p-value), and the y-axis lists the pathway names. This analysis integrates Biological Process (BP), Cellular Component (CC), and Molecular Function (MF) categories, with pathways ranked by significance across these GO categories. The color gradient indicates increasing significance, transitioning from blue to red as -log10(adjusted p-value) increases. The size of the circles represents the number of genes enriched in each pathway.

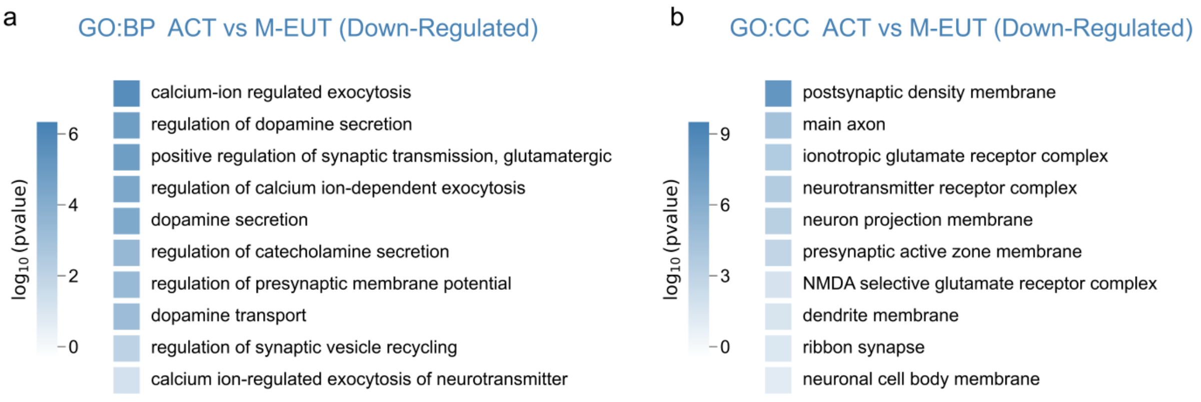

Figure S2. Enrichment plots for the complementary sets of ACT vs. M-EUT and M-DS vs. M-EUT. **a** Gene Ontology-Biological Process (GO-BP) enrichment pathway chart for the genes in the intersection of the complementary sets of ACT vs. M-EUT and M-DS vs. M-EUT. **b** GO-BP enrichment pathway chart for the genes at the intersection of the complementary sets of ACT vs. M-EUT and M-DS vs. M-EUT. Color intensity represents log$_{10}$ (p-value), with darker colors indicating larger log$_{10}$ (p-value) magnitudes.

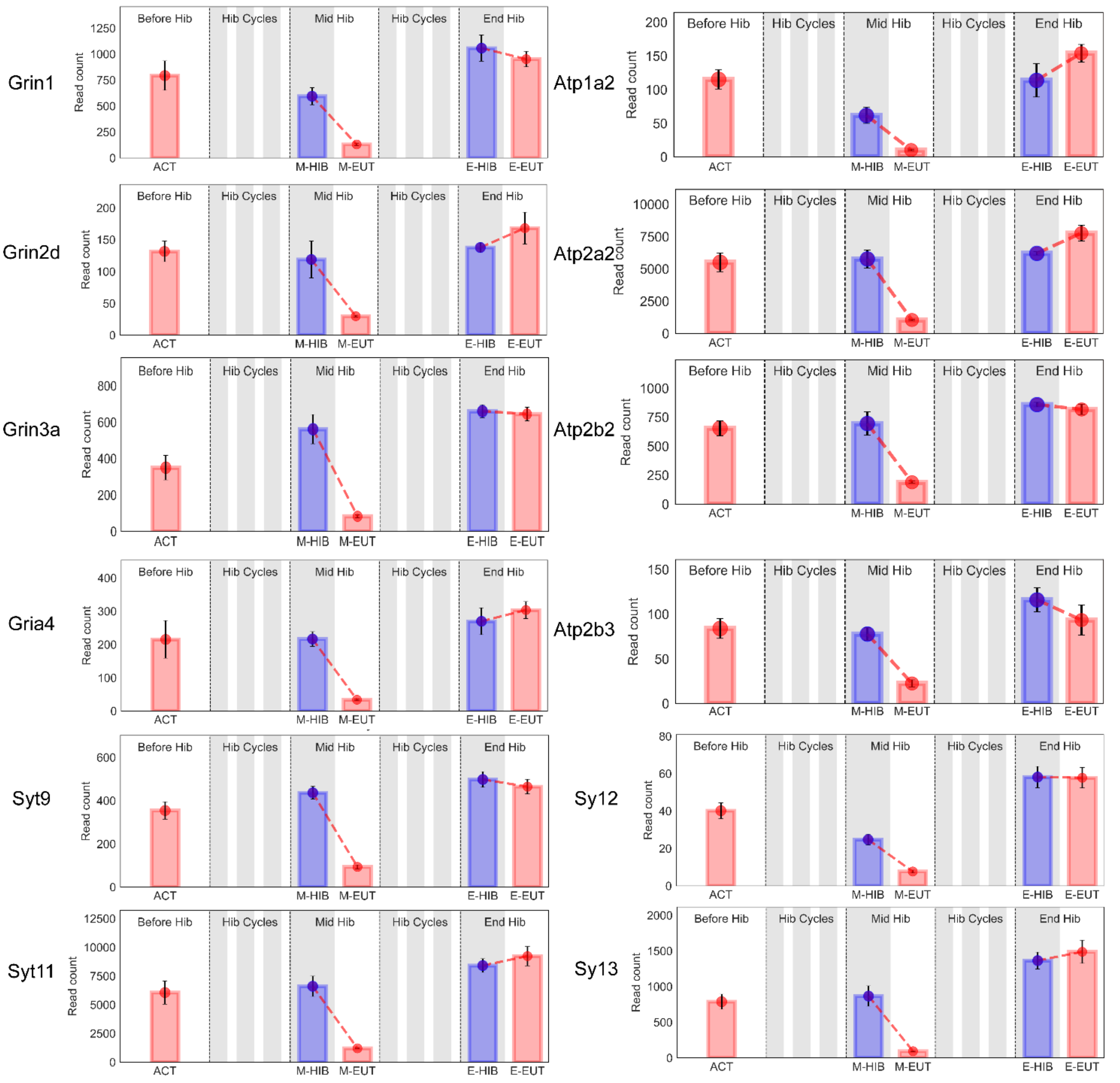

Figure S3. Bar graphs of Grins, Atps, and Syts gene expression in the hypothalamus of T. sibiricus at different time points. Each graph, from left to right, represents the following stages: pre-hibernation (Before Hib), hibernation cycles (Hib Cycles), mid-hibernation (Mid Hib), and end-of-hibernation (End Hib). The color of the sample represents the sleep/arousal state: red for awake and blue for sleeping. Time points are ordered from left to right as ACT, M-DS, M-EUT, E-DS, and E-EUT.

a

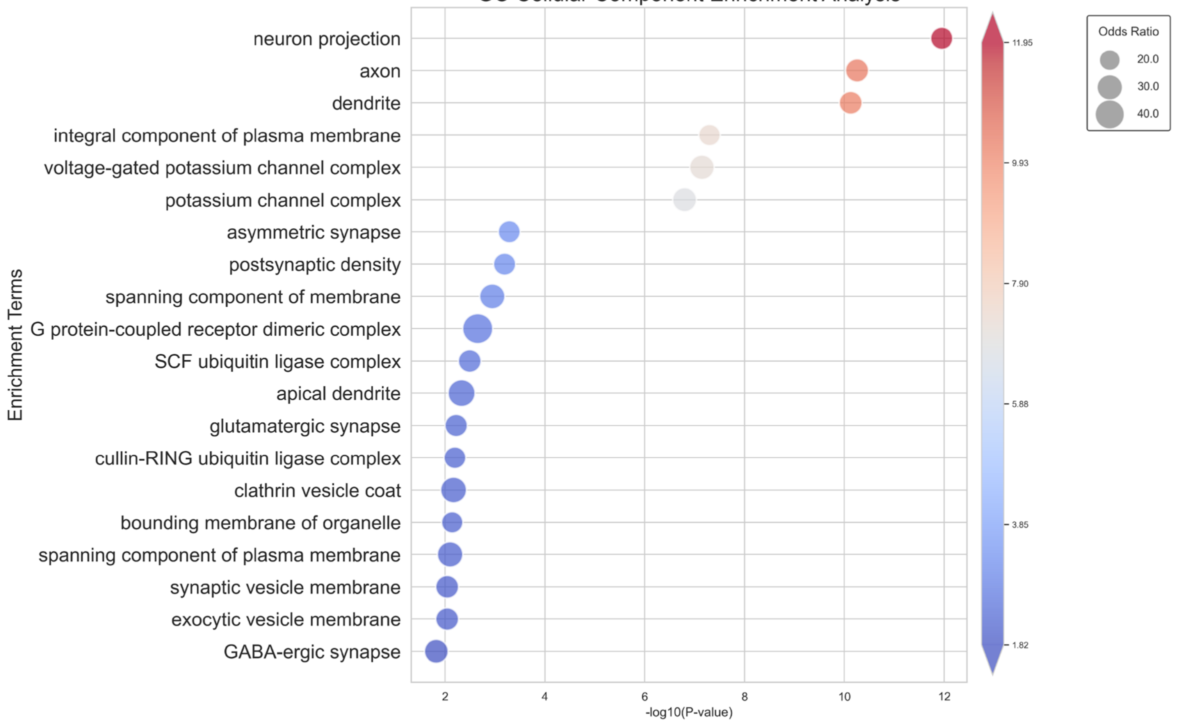

b

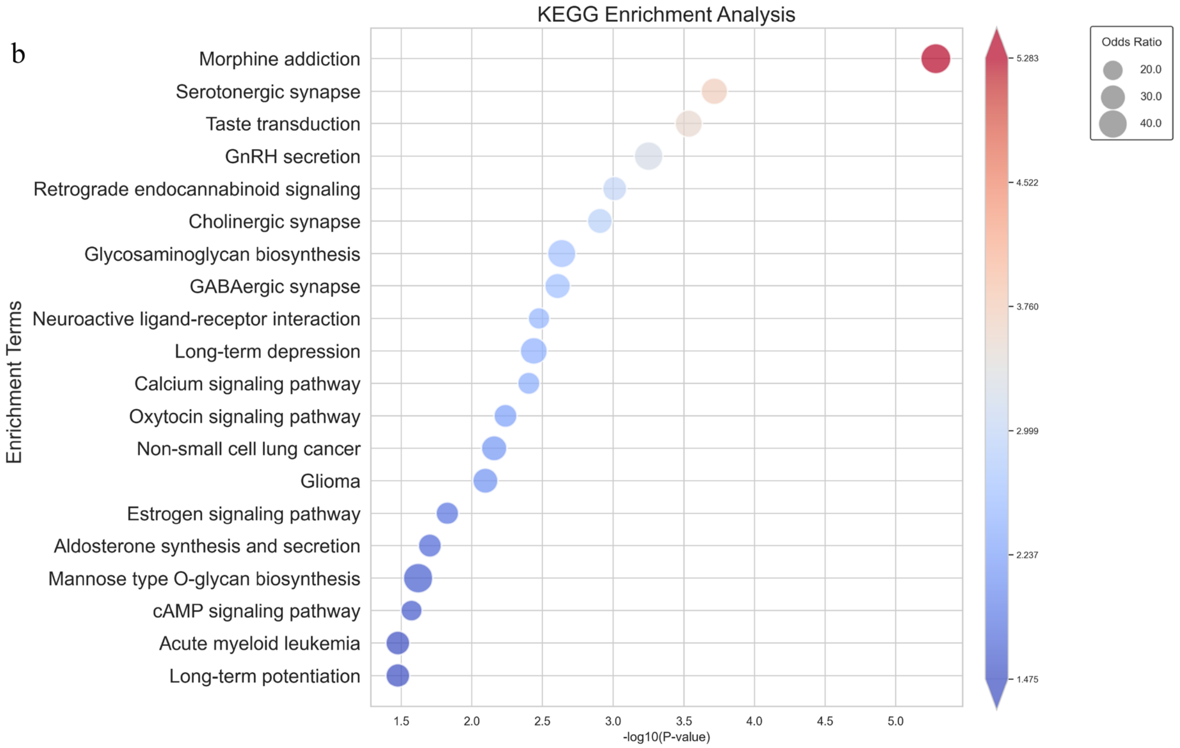

Figure S4. Enrichment analysis of intersected genes in KEGG and GO Cellular Component (GO_CC) pathways. **a** Bubble chart showing the top 20 significantly enriched KEGG pathways, where the size of each bubble represents the odds ratio and the color reflects the -log10(P-value). **b** Bubble chart showing the top 20 significantly enriched GO Cellular Component (GO_CC) pathways, where the size of each bubble represents the odds ratio and the color reflects the -log10(P-value).
